# PAIN2.0: study protocol for a multicentre randomised controlled trial to evaluate the efficacy of a 10-week outpatient interdisciplinary multimodal pain therapy to manage recurrent pain for patients with risk factors of developing chronic pain in Germany

**DOI:** 10.1186/s13063-024-07975-4

**Published:** 2024-02-23

**Authors:** Sandra Meyer-Moock, Daniel Szczotkowski, Leonie Schouten, Frank Petzke, Lena Milch, Beatrice Metz-Oster, Louise Zinndorf, Christian Geber, Greta Hoffmann, Anke Preißler, Ursula Marschall, Felix Rottke, Anja Waidner, André Möller, Thomas Isenberg, Gabriele Lindena, Anne Gärtner, Ulrike Kaiser, Thomas Kohlmann

**Affiliations:** 1 Institute for Community Medicine, University Medicine Greifswald, Greifswald, Germany; 2 Department of Anaesthesiology, University Medical Centre Göttingen, Göttingen, Germany; 3 German Red Cross Pain Centre Mainz, Mainz, Germany; 4 University Pain Centre, University Hospital Carl Gustav Carus, Dresden, Germany; 5 BARMER, Wuppertal, Germany; 6 German Pain Society, Berlin, Germany; 7 CLARA Klinische Und Versorgungsforschung, Kleinmachnow, Germany; 8 University Hospital Schleswig-Holstein/Lübeck, Lübeck, Germany

**Keywords:** Recurrent pain, Complex intervention, Pain and risk factors, Outpatient group therapy, Multimodal interdisciplinary pain management, Public health, Secondary prevention, Mixed models for repeated measures, Health-related quality of life, Randomised controlled trial

## Abstract

**Background:**

Up to 27% of the German population suffers from recurrent or persistent pain (lasting more than three months). Therefore, prevention of chronic pain is one major object of pain management interventions. The aim of this nationwide, multicentre, randomised controlled trial is to evaluate the efficacy of a 10-week ambulatory (outpatient) interdisciplinary multimodal pain therapy (A-IMPT) for patients with recurrent pain and at risk of developing chronic pain. This project was initiated by the German Pain Society (Deutsche Schmerzgesellschaft e.V.) and the public health insurance provider BARMER. It is currently funded by the German Innovation Fund (01NVF20023). The study PAIN2.0 focuses on reducing pain intensity and pain-related disability and investigates whether this intervention can improve physical activity, psychological well-being, and health literacy.

**Methods:**

PAIN2.0 is designed as a multicentre 1:1 randomised controlled trial with two parallel groups (randomisation at the patient level, planned *N* = 1094, duration of study participation 12 months, implemented by 22 health care facilities nationwide). After 6 months, patients within the control group also receive the intervention. The primary outcomes are pain intensity and pain-related impairment, measured as Characteristic Pain Intensity (PI) and Disability Score (DS) (Von Korff), as well as patient-related satisfaction with the intervention. Secondary outcomes are the number of sick leave days, sickness allowance, treatment costs, psychological distress, health-related quality of life, and catastrophizing. The effects of the intervention will be analysed by a parallel-group comparison between the intervention and control groups. In addition, the long-term effects within the intervention group will be observed and a pre-post comparison of the control group before and after the intervention will be performed.

**Discussion:**

Recurrent or persistent pain is common in the German population and causes high costs for patients and society. The A-IMPT aims to improve pain and pain-related impairments in pain patients at risk of chronification, thereby reducing the risk of developing chronic pain with its high socioeconomic burden. This new therapy could easily be integrated into existing therapy programs if positively evaluated.

**Trial registration:**

The trial PAIN2.0 has been registered in the German Clinical Trials Register (DRKS) since 21/11/2022 with the ID DRKS00030773.

## Administrative information

Note: the numbers in curly brackets in this protocol refer to SPIRIT checklist item numbers. The order of the items has been modified to group similar items (see http://www.equator-network.org/reporting-guidelines/spirit-2013-statement-defining-standard-protocol-items-for-clinical-trials/).

**Table:** 

Title {1}	PAIN2.0: study protocol for a multicentre randomised controlled trial to evaluate the efficacy of a 10-week outpatient interdisciplinary multimodal pain therapy to manage recurrent pain for patients with risk factors of developing chronic pain in Germany
Trial registration {2a and 2b}	The trial PAIN2.0 has been registered in the German Clinical Trials Register (DRKS) since 21/11/2022 with the ID DRKS00030773 [1]
Protocol version {3}	Date and version identifier: • Study protocol vs1.0 14/07/2022: Original • Study protocol vs1.1 06/12/2022: Minor changes concerning randomisation • Study protocol vs1.2 19/12/2022: Amendment to ethical and legal aspects The latest version of the study protocol (version 1.2 dating from 19/12/2022) serves as basis of this publication.
Funding {4}	The study is funded by the German Federal Joint Committee’s Innovation Fund (Innovationsausschuss beim G-BA, Postfach 120606, 10596 Berlin, Germany, grant number 01NVF20023) from April 1st 2022 to March 31st 2025.
Author details {5a}	Sandra Meyer-Moock, Institute for Community Medicine, University Medicine Greifswald, Germany Daniel Szczotkowski, Institute for Community Medicine, University Medicine Greifswald, Germany Leonie Schouten, Department of Anaesthesiology, University Medical Centre Göttingen, Germany Frank Petzke, Department of Anaesthesiology, University Medical Centre Göttingen, Germany Lena Milch, German Red Cross Pain Centre Mainz, Germany Beatrice Metz-Oster, German Red Cross Pain Centre Mainz, Germany Louise Zinndorf, German Red Cross Pain Centre Mainz, Germany Christian Geber, German Red Cross Pain Centre Mainz, Germany Greta Hoffmann, University Pain Centre, University Hospital Carl Gustav Carus Dresden, Germany Anke Preißler, University Pain Centre, University Hospital Carl Gustav Carus Dresden, Germany Ursula Marschall, BARMER, Wuppertal, Germany Felix Rottke, German Pain Society, Berlin, Germany Anja Waidner, German Pain Society, Berlin, Germany André Möller, German Pain Society, Berlin, Germany Thomas Isenberg, German Pain Society, Berlin, Germany Gabriele Lindena, CLARA Klinische und Versorgungsforschung, Kleinmachnow, Germany Anne Gärtner, University Pain Centre, University Hospital Carl Gustav Carus Dresden, Germany Ulrike Kaiser, University Hospital Schleswig–Holstein/Lübeck, Germany Thomas Kohlmann, Institute for Community Medicine, University Medicine Greifswald, Germany
Name and contact information for the trial sponsor {5b}	n/a There is no “sponsor” of the trial as it does not include medication or medical device investigations
Role of sponsor {5c}	The funding body has no role in the design of the study and will not have any role during the collection, analysis, interpretation of data, and writing the manuscript.

## Introduction

### Background and rationale {6a}

‘Prevention of chronic pain’ was the theme of the International Association for the Study of Pain’s conference Global Year 2020 (IASP’s Global Year) [2]. However, activities to develop, evaluate and improve diagnostic and treatment options for prevention are still rare. Given the overall burden of chronic pain on individuals and society [3–5], the goal of preventing recurrent pain from becoming chronic should be a high priority for researchers, clinicians, and policy makers.

According to a nationwide survey of German citizens, approximately 27% experience recurrent pain in various locations, 7% experience recurrent pain with functional and somatic limitations, and 3% report recurrent pain with biological and psychosocial limitations [6]. These findings are consistent with other European studies [5].

Currently, available diagnostic and treatment options commonly focus on the treatment and management of chronic pain. However, health care delivery for individuals transitioning from acute to chronic pain is often characterised by overtreatment with medications, invasive treatments (e.g. surgery) and imaging diagnostics, accompanied by undertreatment in the form of early psychosocial and interdisciplinary diagnostics and interventions [7]. These established approaches thus lead to iatrogenic effects (risk factors caused by the health care system, e.g. misinformed care givers or unimodal treatment approaches), enhancing the development of chronic pain forms in general [8]. Preventing individuals from developing impairing chronic pain conditions not only alleviates the burden on those affected but also helps saving limited social and economic resources.

The perspective of prevention regarding pain management comprises three different stages [9]. Primary prevention focuses on identifying and improving maladaptive and non-functional workplaces and lifestyle habits that contribute to the occurrence of pain resulting from the over- or disuse of the body. Secondary prevention aims to maintain or improve physical and/or psychosocial functioning despite recurrent pain while preventing the development of chronic pain conditions. Tertiary prevention addresses individuals with chronic pain, thus reducing the risk of work disability, impaired social participation and the increase of psychological consequences such as depression, anxiety or loneliness. All approaches targeting pain and its management require the involvement of a multidisciplinary team in an integrated approach, ensuring the acknowledgement of the biopsychosocial nature of any kind of pain experience.

The biopsychosocial understanding of pain (either acute or chronic) [10] demands corresponding approaches covering biopsychosocial mechanisms of causing or maintaining recurrent pain experience. Biopsychosocial health care delivery (syn. also interdisciplinary, integrative pain care) includes complex interventions (multimodal), provided by a multidisciplinary team within an integrated team approach (interdisciplinary pain treatment, see IASP Homepage [11]). Key characteristics are equally contributed information and an integrated alignment on diagnoses and treatment components basing on a shared understanding of the individual pain model of the patients using the same language and philosophy.

While biopsychosocial approaches have a well-established history in the treatment and management of chronic pain [12], their development in the context of prevention has been somewhat inconsistent.

Consequently, a concept of interdisciplinary group therapy was developed, piloted and adapted as outlined by Kaiser et al. in 2020 [13]. The intervention’s concept, which falls under the category of secondary prevention, draws from existing evidence regarding individuals with recurrent pain and those at risk of developing chronic pain.

The overall aim of PAIN2.0 is to improve the care of patients undergoing the transition from acute to chronic pain in an outpatient setting. The study involves the implementation of an ambulatory (outpatient) interdisciplinary multimodal pain therapy (A-IMPT) for individuals with persistent or recurrent pain, who, at the time of indication, have risk factors for the development of chronic pain and an increased physical and/or psychosocial disability.

#### PAIN2.0

PAIN2.0 is a consortium project of the Innovation Fund (01NVF20023) with a duration of 36 months.

The consortium leader is the German Pain Society (Deutsche Schmerzgesellschaft e.V.). The consortium partners involved in the project management (PM) are a public health insurance provider (BARMER), an external evaluation institute (Institute for Community Medicine, University Medicine Greifswald), as well as four institutions with profound experience in delivering interdisciplinary multimodal pain therapy (IMPT) (German Red Cross Pain Centre Mainz, University Hospital Carl Gustav Carus Dresden, University Hospital Schleswig–Holstein/Lübeck, University Medical Centre Göttingen).

### Objectives {7}

The main objective of the project is to improve health care delivery for patients suffering from recurrent pain and present risk factors for chronification. Therefore, a therapy adapted to the needs of outpatients with pain is developed. The therapy entails delivering early information on the characteristics of pain, possible risks of chronicity, and the early identification of strategies to mitigate mechanisms that lead to chronicity. The transfer of a biopsychosocial understanding of the disease and specific knowledge about individual and contextual risk-factors influence pain onset equip patients to develop adaptive coping strategies. The ultimate goal is to empower patients to take an active role in managing their pain. The following objectives for an outpatient interdisciplinary multimodal pain therapy are derived:

- Enhancing the sense of control and self-efficacy
- Teaching self-responsibility and building competence in the use of pain reduction techniques
- Introducing a biopsychosocial model
- Providing education about the specificity of pain
- Offering information on psychosocial risk factors

Therefore, the A-IMPT aims to (1) improve the objective and subjective performance as well as the subjective ability to control (physical activity and performance, health literacy, flexible self-regulation) and (2) prevent the development of chronic pain, especially in terms of pain intensity and pain-related impairment, compared to standard care.

The working hypotheses are:

1. Over a 6-month period, participants in the intervention group are expected to report lower pain intensity than those in the control-group.
2. Over a 6-month period, participants in the intervention group are expected to report less pain-related functional disability than those in the control-group.
3. Over a 6-month period, participants in the intervention group are expected to report higher levels of treatment satisfaction compared to those in the control-group.

#### Trial design {8}

**Fig. 1:**
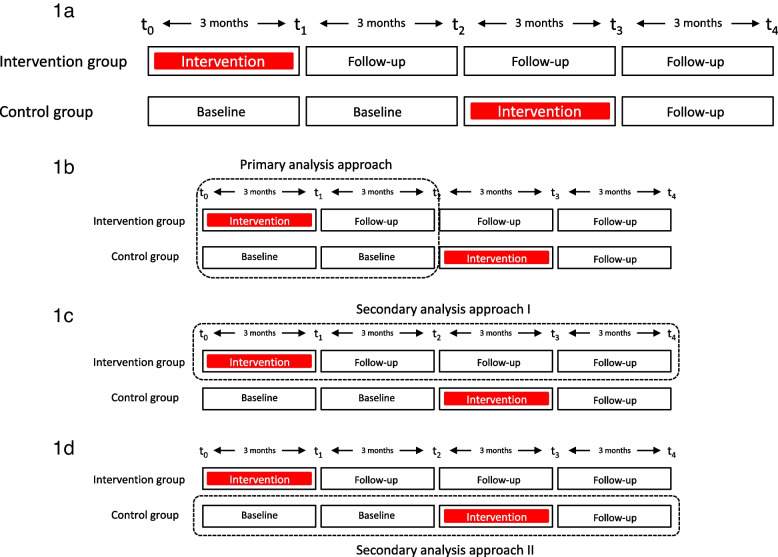
Timeline and analyses approach

The study is designed as a nationwide, multicentre randomised controlled trial (RCT), with two parallel groups to evaluate the efficacy of a 10-week outpatient interdisciplinary multimodal pain therapy (superiority trial). The duration of study participation is 12 months. Patients are enrolled consecutively from January 2023 to November 2023. Following verification of inclusion and exclusion criteria, they will be allocated to either the intervention group or the control group in a 1:1 ratio. The intervention is scheduled to occur between March 2023 and August 2024. Control group patients will receive standard care for 6 months, with the option to participate in the intervention program subsequently. Please refer to Fig. 1 for an overview of the study’s procedural timeline.

Intervention effects will be analysed as follows:

1. Essentially, the intervention effect will be evaluated by analysing the course of the primary and secondary outcomes in the intervention and control groups within the first 6 months (parallel-group comparison, *t*_0_ to *t*_2_) (Fig. 1b).
2. In a secondary analysis approach, the long-term course of the new intervention is examined (*t*_2_ to *t*_4_). Further analyses of the outcome variables at these points are important for evaluating the stability of this effect over time (long-term) (Fig. 1c).
3. The control group receives the intervention in the second half of the study period. The course of the primary and secondary outcomes in the control group after starting the intervention (*t*_0_ to *t*_2_ vs. *t*_2_ to *t*_4_) will be analysed (Fig. 1d). From a methodological perspective, this pre-post comparison can also help mitigating selection biases by increasing the willingness to participate in the study and reducing the drop-out rate within the control group.

## Methods: participants, interventions and outcomes

### Study setting {9}

Participating centres are healthcare institutions (*n* = 22) from all over Germany (distributed in 11 of 16 federal states): 18 centres are located in large cities, 3 centres in medium-sized cities and 1 centre in a rural region. Four centres are outpatient practices, 7 smaller hospitals and 11 larger hospital/university clinics. PAIN2.0 centres are health care institutions in Germany that already offer IMPT or that have the prerequisites for cooperation between the professions required for IMPT according to national scientific recommendations [14, 15]. The participating centres are listed in Table 1.

**Table 1:** List of participating centres

• Georg-August-Universität Göttingen, Universitätsmedizin Göttingen
• DRK-Schmerz-Zentrum Mainz, gemeinnützige Trägergesellschaft Süd-West mbH
• Universitätsklinikum Carl Gustav Carus and er der technischen Universität Dresden, AöR, Universitäts-Schmerz-Centrum Dresden
• AMEOS Klinikum St. Elisabeth Neuburg
• Universitätsklinikum Würzburg
• Universitätsklinikum Freiburg
• Vitos Orthopädische Klinik Kassel gGmbH
• Brüderkrankenhaus St. Josef Paderborn
• Schmerz- und Palliativzentrum Rhein-Main in Wiesbaden
• Westmecklenburg Klinikum Helene von Bülow GmbH Hagenow
• Universitätsklinikum Essen AöR
• Medizinische Hochschule Hannover
• Krankenhaus Bad Oeynhausen der Mühlenkreiskliniken AöR
• Universitätsklinikum Schleswig–Holstein, Campus Lübeck
• Krankenhaus Mörsenbroich-Rath GmbH, Düsseldorf
• Praxis für ganzheitliche Schmerztherapie im Franziskus-Carré Münster
• Universitätsklinikum Heidelberg
• Berufsgenossenschaftliches Universitätsklinikum Bergmannsheil gGmbH Bochum
• Universitätsklinikum des Saarlandes, Homburg
• Asklepios Kliniken Hamburg GmbH—Asklepios Klinik Nord
• Praxis für ganzheitliche Schmerztherapie Dr. Cayemitte-Rückner, Hamburg
• Zentrum für ambulante Rehabilitation GmbH Münster

The centres play a crucial role in the implementation of the new intervention, undertaking vital responsibilities in patient recruitment (including patient information and securing informed consent), overseeing and documenting study-related procedures, collecting data, and transmitting data sets. Each centre receives comprehensive training on study protocols and tasks, both study-related and intervention-related. They actively participate in (online) project meetings and profession-specific intervision and undergo thorough monitoring. Additionally, these centres contribute to regional recruitment efforts, providing support and outreach within their respective areas.

## Eligibility criteria {10}

*Primary* criteria for study inclusion comprise the following:

- New onset of pain (at least 6 weeks ago) or recurrent forms of pain or pain persisting for a longer period
- Pain-related limitations relevant to the patient (e.g. previous sick leave days, interference with daily life, family, leisure, work, and homework) and associated with reduced quality of life
- A risk of developing chronic pain (e.g. pain spread, negative mood, family and work stress factors, avoidance, and avoidance behaviour)

Patients must be at least 18 years old, have sufficient knowledge of the German language in spoken and written form, live in the vicinity of the participating health care facility, and have given their verbal and written consent to participate.

*Primary* criteria for study exclusion comprise the following:

- Clinical signs of a serious illness requiring urgent acute therapy or other serious illnesses (red flags; e.g. severe cardiac insufficiency, tumour)
- A manifest chronic pain condition that has already occurred (e.g. sick leave due to pain for more than 6 months, previous treatment with strong opioids for more than 3 years, previous IMPT)
- A severe and active psychiatric disorder (personality disorder, severe depression, signs of suicidal tendencies)
- An ongoing application for retirement
- A rehabilitation procedure planned for the near future
- Linguistic and/or cognitive impairments

The primary inclusion and exclusion criteria refer to the target population of PAIN2.0. However, there are additional *secondary* inclusion and exclusion criteria that are relevant for group participation and once again describe a subpopulation from the overall target population for inclusion in the PAIN2.0 group programme.

In addition to the general requirement of being able to participate in a 10-week group therapy and residing in close proximity to the facility, profession-specific secondary exclusion criteria were established. These secondary exclusion criteria included medical, physiotherapeutic, and psychological indicators that would limit regular participation in the therapy program and the benefits thereof. They are as follows:

- Medical:
  - Limited physical function for A-IMPT from a medical point of view, health check (check of exclusion criteria, medical)
  - Presence of concomitant diseases that require special monitoring or limit the resilience for the A-IMPT
  - Need for withdrawal treatment that requires continuous monitoring
  - Existing drug dependence (e.g. opioids, benzodiazepines)
  - Presence of an acute pain exacerbation that makes participation in the A-IMPT impossible
- Psychological:
  - Restricted psychological function
  - Restricted social resources in terms of supporting constant group attendance
  - Presence of a manifest, severe psychiatric/psychological diagnosis that primarily indicates psychotherapy (e.g. severe depression, personality disorder)
  - Limited group capacity
  - Restricted motivation to participate regularly
- Physiotherapy:
  - Limited self-care (i.e. daily physical activities, in the sense of managing personal affairs, personal hygiene and mobility)
  - Limited mobility for participation in the A-IMPT
  - Limited physical capacities
  - Pronounced movement-related fear-avoidance behaviour
  - Restricted movement-related competencies
  - Indication for IMPT with higher intensity (“more” than A-IMPT)
  - Need for priority, specific physiotherapy treatment

### Who will take informed consent? {26a}

Informed consent is obtained by the responsible physicians in the centres. They provide comprehensive information about the study and its schedules.

Before asking for consent, the procedure of inclusion contains two steps: primary inclusion criteria will be evaluated by the responsible physician and cover eligibility criteria as described above. Secondary inclusion criteria will be evaluated by the team (physician, physiotherapy and psychology). After fulfilling primary and secondary criteria, patients will be asked to give consent and will be consecutively included in the study.

The participant information materials and informed consent form are available from the corresponding author on request.

### Additional consent provisions for collection and use of participant data and biological specimens {26b}

N/a. There are no additional consent provisions. This trial does not involve collecting biological specimens for storage.

## Interventions

### Explanation for the choice of comparators {6b}

The comparator is the standard healthcare as currently applied in the standard pain therapy in Germany. Patients in the intervention group will receive the new intervention at the beginning of the first 6 months whereas participants in the control group will not receive any trial-related healthcare interventions. However, they are permitted to continue their pre-existing medical, physiotherapeutically and/or psychological (pain-related) therapies, which is often referred to as “treatment as usual”.

**Fig. 2:**
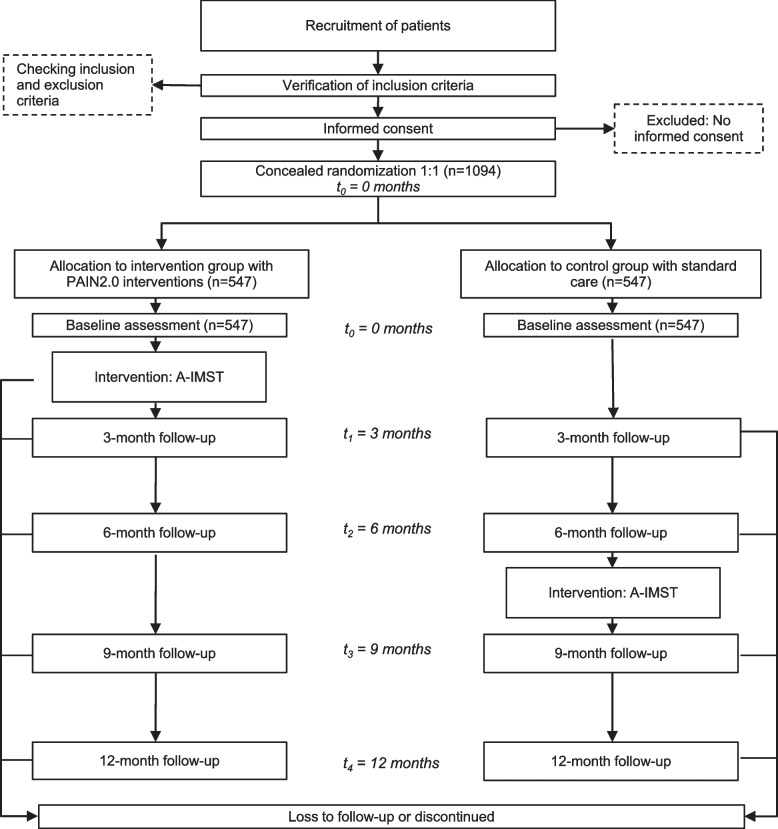
Flow chart of participants

The comparator was chosen to identify the effects of the new intervention and to improve the currently applied pain therapy. Therefore, a comparison to “usual care” is eligible. It is worth noting that patients in the control group also receive interventions after the initial 6 months (Fig. 2; see also Fig. 1).

### Intervention description {11a}

Interdisciplinary multimodal pain therapy [16] is commonly applied in chronic pain treatment. It is described as an integrative therapy approach consisting of somatic, psychotherapeutic, and physiotherapeutic interventions. Physicians, physiotherapists and psychologists collaborate equally in an integrative team approach and regularly coordinate and align on diagnosis, treatment plan and treatment progress. The primary goal of treating chronic pain by IMPT is to restore patient’s subjective and objective functioning and self-control.

In this project, the IMPT is applied to an outpatient setting (A-IMPT) and adapted to the aims for the above-described target population.

**Fig. 3:**
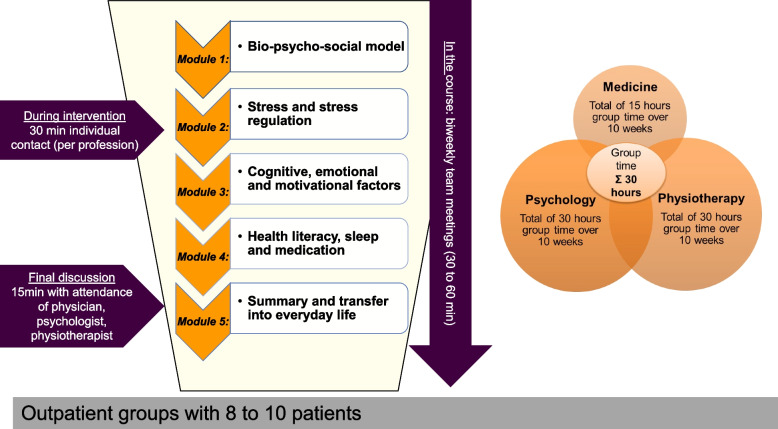
Treatment plan and components of the A-IMPT

The A-IMPT takes place once a week for 3 h in 10 sessions over 12 weeks in groups of 10 patients. It consists of 5 modules in total, each thematically grouped (Fig. 3). There are two sessions within each module, led by the respective professions. Throughout the 10 sessions, psychologists and physiotherapists are each present for a total of 30 h, while the presence of the physicians in the group sums up to 15 h. It is important to note that the content within each module is taught collaboratively by one or more professions.

In the beginning, the general focus is on knowledge transfer, which is progressively expanded through practical exercises and integrated into the patient's background of experience (deepening of problem actualisation and enhancing motivational clarification). Therapeutic interventions (resource activation, problem-solving) are introduced from the beginning to support the transfer into everyday life. Each session ends with practical content (exercises, discussion, task to take home), which is taken up in the following week’s session and leads into the new topic. In this way, patients experience health literacy to maintain a healthy, physically active lifestyle.

In addition to group therapy, all therapists also have the flexibility to offer 30 min of one-on-one time to each patient throughout the 10 weeks. Regular interdisciplinary team meetings are scheduled every two weeks to discuss each patient, lasting 30 to 60 min. A final meeting including the patient and all of the involved healthcare professionals of about 15 to 20 min completes the treatment. It serves to summarise the patient’s experiences, diagnoses, and further recommendations. As a conclusion, a collective final letter is written.

Coordination of group appointments, cancellations and provision of materials is handled by the documentation assistant or the nursing staff.

### Criteria for discontinuing or modifying allocated interventions {11b}

Discontinuation of the study at the participants’ request is possible at any time during participation in treatment or waiting period without giving reasons.

During participation and waiting period, patients continue to have full access to the health care system (i.e. primary care physicians or specialists). Changes in the participants’ medical treatment are assessed during the individual one-on-one contact with the physician as part of the A-IMPT. In the delayed treatment period of the control group, medical changes will be additionally assessed at the face-to-face interim contact (30 min) 3 months after study inclusion.

Reasons for study discontinuation also include any medical conditions that impact the participant's ability to continue participating in the A-IMPT, whether they are related to pain or unrelated medical circumstances (e.g. need for urgent surgery or hospitalisation). Exacerbation of pain requiring intensified pain management (inpatient treatment/hospitalisation) will result in study discontinuation. However, discontinuation is not necessary if the pain exacerbation or new-onset pain can be adequately treated (including surgical intervention or short-term opioid prescription, if necessary) without compromising the ability to participate in the intervention (A-IMPT). Furthermore, physiotherapeutic, and psychological reasons for study discontinuation are documented during the biweekly team meetings. The decision to discontinue the study is always made collaboratively by the treatment team.

### Strategies to improve adherence to interventions {11c}

Before the start of recruitment, the centres receive training in the interventions, study documentation and data management, utilization of assessment instruments, transmission of study documentation to the evaluating institutions, as well as the procedures and obligations regarding data validation. Training is mandatory; furthermore, written and video-based training materials are accessible through an access-restricted website.

A comprehensive monitoring concept has been developed and will be executed by the project administration (Deutsche Schmerzgesellschaft e.V.) in collaboration with consortium partners, including the German Red Cross Pain Centre Mainz, University Hospital Carl Gustav Carus Dresden, University Hospital Schleswig–Holstein/Lübeck, University Medical Centre Göttingen, and the evaluating institution (University Medicine Greifswald). The monitoring aims to oversee the implementation and adherence to the study protocol, provide direct feedback to the centres, and to document any relevant deviations. It commences immediately after the recruitment and inclusion of the first patients.

For participants of the control group, an intermediate medical visit (interim contact) is scheduled after 3 months to prevent dropout. In addition to reassessing the status of the inclusion and exclusion criteria, a brief status report on the patient’s condition is given and the patients should be motivated to attend the later intervention.

The principle of data management and monitoring has been described in a comprehensive data management plan.

### Relevant concomitant care permitted or prohibited during the trial {11d}

Participants are allowed to continue pre-existing pharmacological and non-pharmacological therapies (treatment as usual; see also Sect. 6b) as long as these therapies do not interfere with the primary exclusion criteria (e.g. opioid intake > 6 months).

### Provisions for post-trial care {30}

N/a. No provisions are planned.

### Outcomes {12}

#### Primary outcomes

The primary outcomes are (1) characteristic pain intensity (PI), (2) disability score (DS) for pain-related impairment and (3) patient-related satisfaction. PI and DS are collected at baseline (*t*_0_) and at 3-, 6-, 9- and 12-month follow-up (*t*_1_ to *t*_4_), patient-related satisfaction is measured at 3-, 6-, 9- and 12-month follow-up (*t*_1_ to *t*_4_). The primary evaluation approach covers baseline (*t*_0_) and 6-month follow-up (*t*_2_). Data is collected through a patient questionnaire, which includes the following:

- (1) Characteristic pain intensity and (2) disability score are components of the Von Korff Chronic Pain Grade Scale (CPGS) [17]. The CPGS is a multidimensional measure with 7 items covering the two mentioned dimensions of overall chronic pain severity. The PI score is calculated as the average of three 0–10 ratings for ‘pain right now’, ‘average pain’ and ‘worst pain’, which is then multiplied by 10 to obtain a 0–100 score. The DS score is derived from the average of three 0–10 ratings ‘daily activities’, ‘social activities’, and ‘work activities’, also multiplied by 10 to produce a 0–100 score. The last item covers the disability days. (3) Patient-related satisfaction with the intervention is evaluated using an adapted version of the Patient Satisfaction Questionnaire (ZUF-8) [18, 19], which has been modified slightly to suit the outpatient context. Additionally, a global change item for assessing patient satisfaction is included in the evaluation.

#### Secondary outcomes

The secondary outcomes are (1) psychological distress, (2) health-related quality of life, (3) catastrophizing (primary data) as well as (4) the number of sick leave days, (5) sickness allowance, and (6) treatment costs (secondary data). Primary data is collected through a patient questionnaire at baseline (*t*_0_) and on 3-, 6-, 9-, and 12-month follow-up points (*t*_1_ to *t*_4_), which includes the following:

1. Psychological distress is evaluated using the short form of the Depression, Anxiety and Stress Scale (DASS) [20, 21]. The DASS includes three subscales, each composed of 7 items: depression, anxiety, and stress.
2. Health-related quality of life is measured by the Veterans RAND 12-Item Health Survey (VR-12) [22–25]. This assessment instrument comprises 12 items, which are used to calculate two separate sum scales for physical and mental health. Each sum scale includes all 12 items, with specific weightings applied to the 6 items associated with the physical sum scale and the 6 items linked to the mental sum scale. Higher values on these sum scales indicate elevated levels of either physical or mental health.
3. The Pain Catastrophizing Scale (PCS) [26] examines catastrophizing in regard to pain. The PCS consists of 13 statements covering a range of thoughts and feelings that patients may experience while in pain. The instrument comprises three subscales: rumination, magnification, and helplessness. Higher scores on these subscales indicate a more pronounced level of pain catastrophizing.
  Secondary data of the patients are made available by the participating health insurance BARMER (data source: BARMER scientific Datawarehouse, W-DWH) 6–9 months after treatment delivery and consist of the following:
4. Sick leave days: The dataset contains the number of sick leave days for each BARMER patient included in the study.
5. Sickness allowance: Additionally, the data set provides details about paid sickness allowance for each BARMER patient. These data, combined with sick leave days, form the basis for calculating the short- and long-term social costs related to incapacity to work.
6. Treatment costs: For each BARMER patient included in the data set, comprehensive cost data is available, covering expenses related to inpatient and outpatient care, medication, physiotherapy, occupational therapy, as well as assistive devices.
  If these secondary data are not available, or not available to a sufficient extent, the number of sick leave days, medication, visits to specialists, inpatient facilities, and other medical service providers (e.g. rehabilitation) can be determined from the responses provided by the patient questionnaire.
  The primary evaluation approach covers the period from the baseline to the 6-month follow-up.
  In addition to the primary and secondary outcome measures, process variables and controls for confounders are also collected to examine the effects of the interventions.
7. Therapy expectation is evaluated using the Patient Questionnaire on Therapy Expectation and Evaluation (PATHEV) [27]. Treatment expectation encompasses the patient’s motivation and expectations regarding future treatment. Subscales assess hope for recovery, fear of change and its consequences, and the perceived alignment between the treatment plan and the patient’s initial condition to be treated.
8. Physical activity is assessed using the European Health Interview Survey Physical Activity Questionnaire (EHIS-PAQ) [28–30]. The EHIS-PAQ is a questionnaire designed to evaluate the extent of physical activity in specific public health-related settings. It assesses physical activity related to work, transportation, and leisure in a typical week.
9. Flexible Self-Regulation is evaluated by the Self-Regulation-Inventory (SSI-K3) [31]. The construct of self-control describes conscious action on one’s own responsibility—i.e. the ability to set goals that fit one's personality and to make the decisions that go with them. It also includes the ability to pursue these goals even over obstacles.
10.  Health literacy is measured with two German specific questionnaires for assessing heath literature in this field: ‘Gesundheitskompetenzfragebogen’ (Health Literacy Questionnaire) [32] and ‘Fragebogen zur Erfassung der bewegungsbezogenen Gesundheitskompetenz’ (BGK, Questionnaire for movement-related health competence) [33]. The underlying concept of the Health Literacy Questionnaire is composed of the 3 facets of health literacy: ‘health goals’, ‘confidence to succeed’, ‘coping skills’. The BGK assesses physical activity-related health competence with individual items of a survey instrument based on the PAHCO model. The physical activity-related health competence (PAHCO) model assumes that individuals require three integrated sub-competences to lead a healthy, physically active lifestyle: movement competence, self-regulation competence and control competence. Selected subscales from each questionnaire are used.

Primary and secondary outcomes, additional variables and follow-ups are presented in Table 2.

**Table 2:** Outcomes, additional variables and follow-ups

		**Timepoints/Follow-ups**					
		***t***_***0***_	***t***_***a***_^***a***^	***t***_***1***_	***t***_***2***_	***t***_***3***_	***t***_***4***_
**Outcome variables**							
**Primary**	Characteristic pain intensity	X		X	X	X	X
	Disability score	X		X	X	X	X
	Patient-related satisfaction			X	X	X	X
**Secondary**	Psychological distress	X		X	X	X	X
	Health-related quality of life	X		X	X	X	X
	Catastrophizing	X		X	X	X	X
	Number of sick leave days^b^						
	Sickness allowance^b^						
	Treatment costs^b^						
**Additional variables**							
***Control***	Therapy expectation	X					
***Process***	Physical activity	X	X	X	X		X
	Flexible self-regulation	X	X	X	X		X
	Health literacy	X	X	X	X		X

^a^Additional follow-up for control and process variables, 5th week of intervention

^b^Data is available 6–9 months after treatment delivery

### Participant timeline {13}

**Fig. 4:**
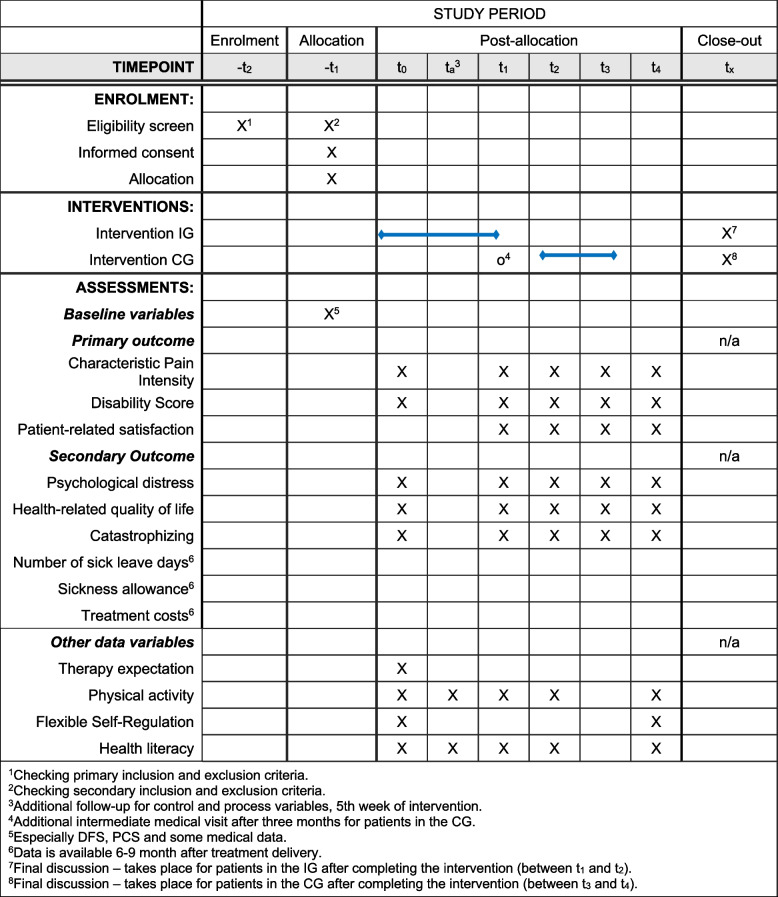
Time schedule of enrolment, interventions and assessments

The complete participant timeline is summarised in Fig. 4. Although details of the timing of enrolment, intervention, and assessments in the PAIN2.0 trial are shown in Figs. 1, 2 and 3 and Table 2.

### Sample size {14}

The sample size was estimated for the analysis of the primary outcomes, which include (1) characteristic pain intensity, (2) disability score and (3) patient-related satisfaction within the first 6 months.

To determine the required number of cases, a simplified multilevel model was employed to examine group differences between standardised mean differences (SRMs) for the three primary outcome measures in the primary analysis approach. The SRM is calculated by dividing the pre-post differences between baseline and follow-up values (*t*_0_ to *t*_2_) by the respective standard deviation of the pre-post differences. The group differences are statistically tested using regression analyses, with the SRMs as the dependent variable and the group membership as the predictor variable.

Power calculation was performed using MLPowSim [34] in a simulation of power to detect group differences. The effect size was defined as the difference between group-specific SRMs, accounting for a residual variance of 0.1%. The differences between the SRMs can therefore be interpreted directly as effect sizes. A value of 0.3 was chosen as the group difference to be detected. These correspond to the size of a minimally significant difference in numerical rating scales in pain research [35]. Type I and type II errors were set at *α* = 0.05 and *β* = 0.20, respectively.

The results of the sample size calculation indicated that a final sample size between 540 and 700 cases is required (statistical power of 80%, 2-sided significance level of 0.05). An expected drop-out of 20% over the 6-month comparison period leads to an increase in the required number of cases to be enrolled initially between *N* = 844 and *N* = 1094. Therefore, it is estimated that approximately *N* = 1355 individuals need to be screened to assess their eligibility criteria.

### Recruitment {15}

Participants are recruited through:

- ▪ The participating health insurance (BARMER)
- ▪ Physicians, psychologists, and physical therapists within regional networks
- ▪ The study centres

The recruitment of insured individuals and patients, as well as their referral to the project, is carried out by the consortium partners project management, the PAIN2.0 centres themselves as well as by cooperating clinicians (general practitioner, specialist, company physicians, other health professionals). Recruitment measure are implemented in a systematic rotation and are repeated in conjunction with new information. The recruitment process spans a period of 5 or 6 months, with the first patient being enrolled in January 2023.

#### Recruitment of referring clinicians

An important factor of early patient care is the timely identification of risk factors by healthcare providers (including general practitioners, specialists, company physicians, orthopaedists, neurologists, physiotherapists, and other health professionals). With the support of the project team (e.g. collection of contact data of potential referrers), PAIN2.0 centres contact patients directly (in person, by telephone, by post), organise information events or collaborate with the regionally established general practitioner training activities and quality circles of various specialist groups (including pain conferences). This network effort is aimed at ensuring the sustainability of the new care service and thus an important component of the project protocol.

Further, the consortium leader Deutsche Schmerzgesellschaft e.V. will contact national networks of potential referring professions and provide information about the study and intervention.

#### Recruitment of patients

Patient recruitment for the A-IMPT primarily relies on press releases and the websites of the participating centres. In addition, BARMER sends informative mail to insured individuals, based on healthcare data suggesting the onset of developing chronic pain. These individuals will have the option to contact BARMER’s specifically trained telephone counselling service (‘teledoctor’). The staff members of teledoctor perform a preliminary check of initial inclusion and exclusion criteria and refer the insured individual directly to the centre or provide the centre’s contact details. Other methods of patient recruitment include direct contact with PAIN2.0 centres through flyers, posters, newsletters, local advertising measures or activities on social media [36]. Patients can also reach out to the centre directly.

#### Recruitment via public relations work

The wider public is informed about PAIN2.0 through the homepage [37], national press releases, publications in specialised media (which not only highlight the unique aspects of the patient group but also describe the innovative A-IMPT care service), outreach to associations, medical associations and businesses as well as advertising on social media platforms. These initiatives are coordinated by the consortium partners project management. Interested referring clinicians and patients can contact the PAIN2.0 centres directly.

In preparation for recruitment, numerous recruitment options were presented to the centres in video conferences and sufficient materials for implementation were handed out (including cover letters, webinars, regional press releases). During the recruitment phase, support and exchange were offered to the centres in video conferences or in the context of individual centre support. To ensure the consistency of project communications, the project team developed text modules that were tailored to different target groups including patients, the professional public, and the general public. These modules were applied in a standardised manner.

## Assignment of interventions: allocation

### Sequence generation {16a}

Lists of randomised blocks of varying lengths were generated by the evaluating institute for each participating centre and implemented in the PAIN2.0 database.

### Concealment mechanism {16b}

Lists of randomised blocks used in the external randomisation were not known to the recruiting centres at the time of patient enrolment.

### Implementation {16c}

Patients are enrolled by physicians who verify the primary inclusion and exclusion criteria.

Those patients who fulfil both the primary and secondary inclusion criteria and having no reasons for exclusion are consecutively included in the study.

Patients providing informed consent were randomly assigned to the intervention or control group by the medical documentarians during study enrolment.

## Assignment of interventions: blinding

### Who will be blinded {17a}

N/a. Blinding is not possible.

### Procedure for unblinding if needed {17b}

N/a. There is no blinding.

## Data collection and management

### Plans for assessment and collection of outcomes {18a}

Prior to the start of recruitment, the participating centres will receive training on the interventions, the utilization of assessment instruments, handling the study documentation, forwarding of the study documentation to the evaluating institutions, and the process and obligations for data validation.

At the time of enrolment (i.e. before *t*_0_), patients will be asked to complete the standard questionnaire of the German Pain Society (DSF) [38]. In addition, medical data is assessed. Further data collection is done through questionnaire surveys at baseline and at 3-, 6-, 9-, and 12-month follow-ups. In addition, a process evaluation questionnaire is to be completed in the fifth week of the intervention (Fig. 4).

Data is archived in a PAIN2.0 database, which contains all documentation.

Monitoring is conducted by the central project coordinator (Deutsche Schmerzgesellschaft e.V.), the consortium partners German Red Cross Pain Centre Mainz, University Hospital Carl Gustav Carus Dresden, University Hospital Schleswig–Holstein/Lübeck, University Medical Center Göttingen, and the evaluating institution (University Medicine Greifswald).

Questionnaires are available at the German Pain Society (Deutsche Schmerzgesellschaft e.V.).

### Plans to promote participant retention and complete follow-up {18b}

To promote participant retention, several procedures have been introduced. Participants in the intervention group receive one individual contact with each professional group during the 10-week therapy. These individual contacts last 30 min each and can be split into two appointments of 15 min each if necessary. Participants of the control group receive an additional intermediate medical visit after 3 months to prevent dropout. The questionnaire response is monitored through the database. If a questionnaire is not returned on time, two follow-up actions are taken. Patients who do not regularly attend the interventions will be contacted by the study centre. Reasons for discontinuation are documented.

A list of outcome data will be compiled for participants who discontinue or deviate from the intervention protocols. This will include detailed information on participants who drop out of the intervention or deviate from the study protocol.

### Data management {19}

Data collection is paper based. The questionnaires are sent out to the patients by post, completed by them and returned to the study centre in self-addressed stamped envelopes. The centres send the questionnaires by (registered) mail to the evaluation institute (University Medicine Greifswald). Data entry is carried out by trained student assistants into a password-protected database according to a codebook. Electronic data entry forms and range checks ensure that only valid values can be entered. Double data entry is made for 10% of the questionnaires. Agreement on data entry is checked. The principle of data management and data protection has been described in a comprehensive data management plan.

### Confidentiality {27}

The following patient data are collected and processed in the centres:

- ▪ Administrative data (e.g. name, date of birth, health insurance company, and the name of the responsible study doctor) only remain in the centres and are used for medical care according to the study conditions and for the organisation of follow-up interviews and group procedures. These data must be retained for a period of 10 years. The list remains at the centre and is the only link between pseudonymised patient ID and the health insurance data.
- ▪ All other information on patient care and evaluation is entered into the PAIN2.0 database exclusively in pseudonymised form, following the written consent of the study participants. In the PAIN2.0 database, the pseudonymised patient data for each centre are presented in separate overviews, with data from other centres being inaccessible.

Questionnaire data for evaluation is entered into the PAIN2.0 database exclusively in pseudonymised form.

### Plans for collection, laboratory evaluation and storage of biological specimens for genetic or molecular analysis in this trial/future use {33}

N/a. There are no plans for collection, laboratory evaluation and storage of biological specimens for genetic or molecular analysis in the current trial and future use in ancillary studies.

## Statistical methods

### Statistical methods for primary and secondary outcomes {20a}

According to the statistical design, mixed models for repeated measures (MMRM) [39] will be applied to analyse the primary and secondary outcome variables, considering the multi-level data structure with centre, patient, and measurement time levels. These models are well-suited for the analysis, including random effects for the regression constant (random intercept model) at the patient level and, if necessary, also at the centre level.

In the regression analyses, the values of the target variables at times t_1_ to t_4_ serve as the dependent variables. To describe the effect of the intervention, the characteristics of the dependent variable at time t_0_, group membership, time of measurement, interaction effects of group membership, and time of measurement are used as predictor variables [39, 40]. When using these models, it is possible to include further predictor variables in the analysis. This can be of interest if, despite randomisation, relevant differences occur between the study arms and must be statistically controlled, or if the effects of covariates (e.g. of age or other patient characteristics) are to be analysed. Through appropriate model specification, all hypotheses of interest can be tested with these analysis methods.

The target variables to be investigated are predominantly continuous data for which linear regression models can be used. However, the family of (generalised) mixed models also allows analogous evaluations for other types (ordinal or categorical) of dependent variables.

In the context of the analysis of days of incapacity to work or cost data, it may furthermore be necessary to use special analysis methods for data with an asymmetric distribution or with heavily populated zero cells (“zero-inflated”).

Statistical significance tests are performed with an α-level of 0.05 (two-sided). In the confirmatory analysis of the primary outcome measures, a Bonferroni adjustment for multiple testing (3 outcome measures, 2 follow-up times) is applied. No such adjustment is used in the exploratory analyses of the secondary outcomes.

### Interim analyses {21b}

N/a. Interim analyses are not planned.

### Methods for additional analyses (e.g. subgroup analyses) {20b}

#### Level of centres

Additional investigations will evaluate existing process and structural parameters as required by the study protocol (e.g. consistency in team collaboration, availabilities of complete staff, aspects of coordination treatment groups and plans, qualification of staff, perceived quality of interaction between staff members while conducting group or team meetings etc.).

#### Patient level

Parameters for evaluating the delivery of intervention will be gathered during the treatment period (process parameters, see above). Satisfaction of patients with group delivery and perceived relationship and support by care givers will be evaluated additionally.

Subgroup formation is planned primarily for the centre level. Centres will be grouped according to local parameters (size of community, local health care delivery and networks in terms of pain management), and will consider the process and structural parameters of the centre (main delivery) and delivery of the study intervention.

### Methods in analysis to handle protocol non-adherence and any statistical methods to handle missing data {20c}

The family of (generalised) mixed models also allow the handling of missing follow-up data. If necessary (e.g. in case of high drop-out-rates), appropriate methods of (multiple) imputation of missing values will be employed.

### Plans to give access to the full protocol, participant level-data and statistical code {31c}

The full protocol is available online at DRKS [1], the statistical code will be provided in the end of the study upon reasonable request. Due to existing data protection rules, no access to participant-level dataset is planned.

## Oversight and monitoring

### Composition of the coordinating centre and trial steering committee {5d}

PAIN2.0 is a consortium project; consortium leader is the German Pain Society (Deutsche Schmerzgesellschaft e.V.) in collaboration with the health insurance company BARMER. The consortium partners involved in the project management (PM) are BARMER, an external evaluation institute (Institute for Community Medicine, University Medicine Greifswald), as well as four institutions with extensive experience in interdisciplinary multimodal pain therapy (IMPT): German Red Cross Pain Centre Main, University Hospital Carl Gustav Carus Dresden, University Hospital Schleswig–Holstein/Lübeck, University Medical Centre Göttingen, University Medicine Greifswald.

The consortium is responsible for the project conception and implementation, the organisation of centre and patient recruitment, support in local network formation as well as the monitoring of the study.

Consortium partners deliver the intervention and fulfil study-related tasks (PAIN2.0-Centers, see Table 1).

An annual Advisory Board consisting of representatives of various committees of the German Pain Society is appointed to advise on the conduct of the study.

### Composition of the data monitoring committee, its role and reporting structure {21a}

The consortium project management is responsible for the project monitoring of the study. It consists of the consortium partners German Pain Society (consortium leader), University Medical Centre Göttingen, German Red Cross Pain Centre Mainz, University Hospital Carl Gustav Carus Dresden, University Hospital Schleswig–Holstein/Lübeck, University Medicine Greifswald, BARMER. All monitoring activities take place independent from the sponsor.

### Adverse event reporting and harms {22}

N/a. The occcurrence of adverse events is not expected in this trial.

### Frequency and plans for auditing trial conduct {23}

Monitoring and auditing a multicentre study with a complex intervention presents unique challenges. The monitoring concept of PAIN2.0 encompasses two key areas:

1. Monitoring of case number achievement, recruitment, and admission according to study protocol and
2. Monitoring the implementation of the provided intervention and follow-up surveys according to the study protocol.

One of the objectives of the monitoring is to ensure the implementation purity of the project protocol in each participating PAIN2.0 centre, considering the different local conditions and prerequisites. In addition, the monitoring also serves to ensure treatment purity as a prerequisite for the strength of the intervention, including the hypothetically assumed effects. The third objective involves the collection and evaluation of the facilities’ experiences in recruiting suitable patients as well as in implementing the new care service, which are of high importance for the subsequent possible roll-out. For each area, the following 5 levels of monitoring are currently envisaged:

- Continuous data monitoring (monthly, individually for each centre): The documentation as well as the implementation of the study (inclusion and A-IMPT) will be monitored via criteria defined in the project team for implementation purity (about the project protocol, including checking primary and secondary inclusion and exclusion criteria) and treatment purity (including implementation of the A-IMPT; reviewing data base). In the event of a relevant deviation from the project protocol, the responsible PAIN2.0 centre will be contacted and will undergo further monitoring.
- Individual audit reports (monthly to quarterly, individual for each centre): During the recruitment phase (January 2023 to November 2023), there will be a monthly individual centre report on caseload management for the PAIN2.0 centres including selected data on implementation and admission purity (retrieved from data base; including caseload, referral pathways, patient characteristics, randomisation). During treatment delivery (March 2023 to August 2024), a quarterly audit report will be provided to reflect intervention purity according to the study protocol.
- Ongoing PAIN2.0 Talks/Ongoing Video Conferences (monthly, online): All centres are invited to participate in one-hour profession-specific talks. The main goal of these monthly meetings is to provide information and resolve any questions or obstacles. The discussions primarily focus on admission and later in the process on the delivery of the health care intervention. A regular protocol is sent out to all members of all centres after completed talk-rounds.
- Visit audits (once for each centre after completing study enrolment and admission): A single visit to each centre is planned to prepare for data delivery after completing the recruitment phase.
- Final audit (twice; one after completed admission/enrolment, one after completing intervention delivery; individually for each centre; online): Monitoring of data entry, quality of data and the compliance of data protection law are focus of the final audits. Reports provide feedback of open or pending tasks of the centres to close data acquisition and management, allowing the start of data delivery to the evaluation institute and to the project team for further/final analyses.

### Plans for communicating important protocol amendments to relevant parties (e.g. trial participants, ethical committees) {25}

Protocol modifications (e.g. changes to eligibility criteria, outcomes, analyses) to relevant parties (e.g. trial participants, ethical committees, investigators, institutional review board, trial participants, trial registry) are communicated by the study coordinating centre (Deutsche Schmerzgesellschaft e.V.) and will be published in the preregistration of this study [1].

### Dissemination plans {31a}

The study protocol is available as open access publication in agreement with SPIRIT [41, 42] and CONSORT [43] criteria. The final trial dataset will primarily be available for the data centre of the project and the independent evaluation institution (University Medicine Greifswald).

Data protection regulations restrict the use of the trial data to purposes and by institutions being agreed upon by participants who have provided written informed consent. Any queries regarding data availability can be forwarded to the corresponding author. The trial results will be made available by scientific publication and reported to the funding body.

Authorship eligibility guidelines for publishing peer-review journals will be applied.

After the study is completed, the main results of the study will be presented in a layman’s summary on the project website.

## Discussion

Recurrent or persistent pain is a widespread issue in the German population, imposing significant costs on individuals and society due to physical and psychosocial impairment. PAIN2.0 is designed to improve the health of individuals with recurrent pain and associated risk factors, subsequently reducing the costs incurred.

Intensive forms of therapy or management programs for chronic pain have already been shown to be cost-effective for chronic pain [44]. Thus, long-term cost reductions can be expected by preventing the occurrence of severe physical and psychosocial impairments and avoiding chronic courses of pain. The study results of PAIN2.0 alongside the economic feasibility analyses, serve as the foundation for integrating outpatient interdisciplinary pain therapy to improve recurrent pain into standard care.

The fact that the study involves a complex intervention is a potential limitation. An equally complex monitoring concept attempts to ensure the comparability of the intervention to be carried out. In addition, regular data monitoring and monitoring of process and structural quality ensure compliance with the study protocol.

Overall, the new form of care could be included into standard practice pending convincing results and approval from the German Federal Joint Committee.

## Trial status

Protocol version number: study protocol version 1.2 dating from 19/12/2022

Start of recruitment: 01.01.2023

End of Recruitment: 30.11.2023

## Data Availability

The study protocol is available as open access publication in agreement with SPIRIT [41, 42] and CONSORT [43] criteria. The final trial dataset will primarily be available to the independent evaluation centre (UMG) and the German Pain Society. Data protection regulations restrict the use of the trial data for purposes and by institutions being agreed on by participants providing written informed consent. Any queries regarding data availability can be forwarded to the corresponding author. The trial results will be made available by scientific publication and reported to the funding body. Authorship eligibility guidelines for publishing peer-review journals will be applied.

## Abbreviations

A-IMPT: Ambulatory (outpatient) interdisciplinary multimodal pain therapy
BGK: Questionnaire for movement-related health competence
CPGS: Chronic Pain Grade Scale
DASS: Depression, Anxiety and Stress Scale
DRKS: German Clinical Trials Register
DS: Disability score
DSF: German Pain Questionnaire (Deutscher Schmerzfragebogen)
EHIS-PAQ: European Health Interview Survey Physical Activity Questionnaire
EQ-5D-5L: EuroQol questionnaire, 5 dimensions, 5 level
HRQoL: Health-related quality of life
IASP: International Association for the Study of Pain
IMPT: Interdisciplinary multimodal pain therapy
PAHCO: Physical activity-related health competence
PATHEV: Patient Questionnaire on Therapy Expectation and Evaluation
PCS: Pain Catastrophizing Scale
PI: Characteristic pain intensity
RCT: Randomised controlled trial
SSI-K3: Self-Regulation-Inventory
UMG: University Medicine of Greifswald
VAS: Visual analogue scale
VR-12: Veterans Rand-12 items
W-DWH: BARMER scientific Datawarehouse
ZUF-8: Patient Satisfaction Questionnaire
